# Molecular Design Strategy of π‐Conjugated Polymers for Efficient Visible‐Light‐Driven Photoelectrocatalytic O_2_ Reduction to H_2_O_2_ Production

**DOI:** 10.1002/cssc.202502396

**Published:** 2026-03-01

**Authors:** Riku Sawada, Hitoshi Kasai, Kouki Oka

**Affiliations:** ^1^ Institute of Multidisciplinary Research for Advanced Materials Tohoku University Sendai, Miyagi Japan; ^2^ Carbon Recycling Energy Research Centre Ibaraki University Hitachi, Ibaraki Japan; ^3^ Deuterium Science Research Unit Centre for the Promotion of Interdisciplinary Education and Research Kyoto University Yoshida, Sakyo‐ku, Kyoto Japan

**Keywords:** catalysis, electrochemistry, hydrogen peroxide, π‐conjugated polymers, sustainable chemistry

## Abstract

Toward sustainable hydrogen peroxide (H_2_O_2_) production, photo(electro)catalytic oxygen (O_2_) reduction/H_2_O_2_ production has attracted increasing attention. Recently, we have found that a thin film of the π‐conjugated polymer, poly(1,4‐bis(2‐thienyl)benzene) (**PBTB**), exhibits exceptionally high (photo)electrocatalytic activity for O_2_ reduction/H_2_O_2_ production. To achieve higher photoelectrocatalytic activity and efficient visible‐light‐driven photoelectrocatalytic H_2_O_2_ production, we investigated the molecular design related to the highest occupied molecular orbital (HOMO) energy level (*E*_HOMO_) of these polymers. We designed and synthesized poly(1,4‐bis(2‐thienyl)naphthalene) (**PBTN**), in which replacing the phenyl unit of **PBTB** with a naphthalene unit—a stronger electron‐withdrawing group—and increasing the polymer chain twist angle selectively deepened *E*_HOMO_ relative to **PBTB**. The degree of *E*_HOMO_ deepening quantitatively affected the onset potential of **PBTN**. Under visible‐light irradiation and 0 V vs. Ag/AgCl, the **PBTN** thin film achieved a high O_2_ reduction/H_2_O_2_ production rate (1.11 × 10^3^ ${\mathrm{mmol}}_{H_{2}O_{2}}$/g_photoelectrocatalyst_), 1.47 times higher than that of **PBTB**, with excellent Coulombic efficiency (99%) and selectivity (99%). The onset potential of **PBTN** for visible‐light‐assisted O_2_ reduction enabled a photocatalytic H_2_O_2_ production setup. Upon visible‐light irradiation, this setup achieved a high photocatalytic O_2_ reduction/H_2_O_2_ production rate of 128 ${\mathrm{mmol}}_{H_{2}O_{2}}$/g_photocathode_. These results clearly demonstrate the tunability of the photoelectrocatalytic activity of π‐conjugated polymers through *E*_HOMO_‐related molecular design.

## Graphical abstract

This study demonstrated the high tunability of the photoelectrocatalytic activity of π‐conjugated polymer thin films for oxygen (O_2_) reduction/hydrogen peroxide (H_2_O_2_) production through molecular design. The degree of HOMO energy level deepening quantitatively affected the onset potential for light‐assisted O_2_ reduction toward more positive values, enhancing the photoelectrocatalytic H_2_O_2_ production rate.

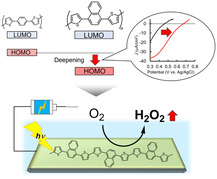

## 1 Introduction

Hydrogen peroxide (H_2_O_2_) is a representative oxidizing agent and is environmentally friendly, as it produces only water (H_2_O) and oxygen (O_2_) after use. H_2_O_2_ plays an important role in various applications, including pulp bleaching [1], chemical synthesis (such as for propylene oxide) [2], wastewater treatment [3], and disinfection [4]. The global production volume of H_2_O_2_ has been steadily increasing, from 5.50 Mt per year in 2015 to 6.55 Mt in 2024 [5, 6].

Currently, more than 95% of H_2_O_2_ is produced by the auto‐oxidation (**AO**) method, which uses anthraquinone derivatives [7]. The **AO** method allows for large‐scale production and remains one of the most cost‐effective methods for producing H_2_O_2_ compared to other currently competing methods [8]. However, it has several drawbacks, such as the use of toxic organic solvents (e.g., benzene), reliance on rare and expensive palladium catalysts, and the generation of large amounts of waste solvents to remove byproducts derived from anthraquinone derivatives [9, 10, 11], making it far from a green and sustainable technology. In addition, the **AO** method requires large‐scale plants for multiple production steps (hydrogenation, oxidation, extraction, and distillation), usually constructed near oil refineries (commonly far from consumption sites) to utilize low‐cost hydrogen gas [11]. These requirements necessitate additional processes for efficient transport of H_2_O_2_ aqueous solution, such as concentrating the solution to 35–70 wt% (which poses an explosion risk), adding stabilizers, and performing further purification to remove stabilizers. As most applications require just low‐concentration H_2_O_2_ aqueous solutions (e.g., 0.1–3 wt%) [12, 13], these processes are inefficient. For a sustainable society, an industrial method that minimizes environmental impact and enables facile H_2_O_2_ production close to consumption sites is urgently required [14].

In this context, H_2_O_2_ production methods that utilize light and/or electrical energy to reduce O_2_ from the air using an appropriate catalyst have attracted significant attention [15, 16, 17]. This approach is green and sustainable because it can produce H_2_O_2_ from O_2_ and H_2_O, which are abundant resources on the Earth, while using renewable energy sources such as sunlight [18] and producing almost no waste throughout the process. Photo(electro)catalysts for O_2_ reduction/H_2_O_2_ production, which are essential for this technology, have been extensively investigated to achieve high activity, selectivity, stability, and scalability [17].

As photo(electro)catalysts for H_2_O_2_ production, inorganic materials such as metal semiconductors (TiO_2_, ZnO, BiVO_4_) and carbon materials have been primarily reported [19, 20]. In particular, as Y. Shiraishi discovered in 2014 that graphitic carbon nitride (g‐C_3_N_4_) functions as a photocatalyst for O_2_ reduction/H_2_O_2_ production [21], carbon materials have been intensively studied owing to their composition of earth‐abundant elements (C, N, O, H) and excellent stability [22]. Although the catalytic performance of carbon materials can be improved through the introduction of structural defects and elemental doping [17, 23], their photocatalytic H_2_O_2_ production rate remains lower than approximately 5.0 ${\mathrm{mmol}}_{H_{2}O_{2}}$/g_photocatalyst_ h. Recently, organic materials with high molecular design flexibility, such as covalent organic frameworks (COFs), have attracted attention, with moderate photocatalytic H_2_O_2_ production rates of approximately 10 ${\mathrm{mmol}}_{H_{2}O_{2}}$/g_photocatalyst_ h being reported [24, 25, 26]. However, as COFs are cross‐linked, they exhibit low solubility in most solvents and are typically obtained as microcrystalline powders, making film formation and large‐scale applications difficult [27].

More recently, π‐conjugated polymers have emerged as promising (photo)electrocatalysts for O_2_ reduction/H_2_O_2_ production [28, 29, 30]. These π‐conjugated polymers have significant advantages over conventional (photo)electrocatalysts, including diverse molecular design options through organic synthesis, the ability to form films via various coating methods [31, 32, 33, 34], a simplified single‐layer architecture functioning as both light absorber and catalyst, high durability (lasting from several days to weeks) [28, 29], and extremely high Coulombic efficiency and selectivity (>95%) for O_2_ reduction/H_2_O_2_ production as photoelectrocatalysts [28, 29, 30]. For example, we previously demonstrated the high‐purity π‐conjugated copolymer of thiophene and phenylene, poly(1,4‐bis(2‐thienyl)benzene) (**PBTB**), synthesized by iodine‐vapor‐assisted polymerization method, [29, 31, 35, 36, 37] as a (photo)electrocatalyst with a high photoelectrocatalytic H_2_O_2_ production rate of around 1.0 × 10^3^ ${\mathrm{mmol}}_{H_{2}O_{2}}$/g_photoelectrocatalyst_ h. Combining a **PBTB** thin film with an H_2_O oxidation/O_2_ production electrocatalyst enables the combined setup (photocatalyst) to achieve a high photocatalytic O_2_ reduction/H_2_O_2_ production rate exceeding 100 ${\mathrm{mmol}}_{H_{2}O_{2}}$/g_photocatalyst_ h under visible‐light irradiation [29]. These results demonstrate the high potential of high‐purity π‐conjugated polymers as (photo)electrocatalysts. However, the relationship between the molecular structure of π‐conjugated polymers and their catalytic performance remains unclear.

In this work, we aimed to clearly demonstrate the advantages of π‐conjugated polymers over other materials by developing a molecular design strategy for tuning photoelectrocatalytic activities (e.g., the onset potential) to achieve further enhancement. Specifically, we focused on the highest occupied molecular orbital (HOMO) energy level (*E*_HOMO_) of π‐conjugated polymers, which was expected to influence their photoelectrocatalytic activity (e.g., the onset potential).

## 2 Results and Discussion

### 2.1 Preparation and Characterization of PBTN Thin Film

**FIGURE 1:**
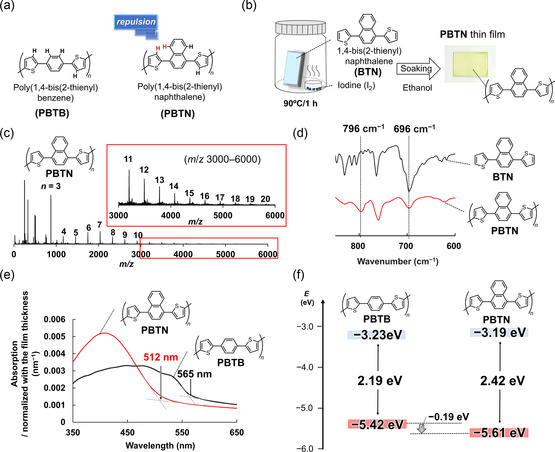
(a) Chemical structure of PBTB and PBTN. (b) Schematic illustration of the iodine‐vapor‐assisted polymerization procedure for the PBTN thin film. The image shows the PBTN thin film formed on a glass plate. (c) MALDI‐TOF MS spectrum of PBTN. (d) FT‐IR spectra of PBTN (red trace) and BTN (black trace) normalized by the peaks at 1380 cm^−1^, corresponding to the C—C stretching vibration of naphthalene units [42]. (e) UV‐Vis absorption spectra of PBTN thin film and PBTB film normalized to film thickness. (f) HOMO/LUMO energy levels and energy gaps of PBTB and PBTN.

We aimed to synthesize π‐conjugated polymers with a selectively deepened *E*_HOMO_ of **PBTB**, which has been reported to exhibit a high (photo)electrocatalytic activity [29], and therefore explored suitable monomers. First, we sought to deepen only *E*_HOMO_ by introducing electron‐withdrawing groups into 1,4‐bis(2‐thienyl)benzene (**BTB**), a monomer of **PBTB**. However, introducing electron‐withdrawing groups typically deepens both *E*_HOMO_ and the lowest unoccupied molecular orbital (LUMO) level (*E*_LUMO_) [38]. As shown in Table S1, quantum chemical calculations also indicated similar trends for **BTB** derivatives containing common electron‐withdrawing groups (e.g., −COOH and −F) on the phenylene unit of **BTB**. Then, we focused on the fact that as the twist angle of the polythiophene chain increases, the π‐orbital overlap within the polymer decreases, resulting in a deeper *E*_HOMO_ and a shallower *E*_LUMO_ [39]. We hypothesized that both introducing electron‐withdrawing groups and increasing the twist angle of the π‐conjugated polymer chain would allow us to deepen only the *E*_HOMO_ without significantly altering *E*_LUMO_. Based on this hypothesis, we designed poly(1,4‐bis(2‐thienyl)naphthalene) (**PBTN**), in which the phenylene unit in **PBTB** was replaced with a naphthalene unit. The naphthalene unit is a stronger electron‐withdrawing group than that of the phenylene unit [40, 41], and the repulsion between hydrogen atoms on the naphthalene and thiophene units, as shown in Figure 1a, was expected to increase the twist angle of the polymer chain (Figures 1a and S1).

As shown in Figure 1b, the **PBTN** thin film was prepared by iodine‐vapor‐assisted polymerization of 1,4‐bis(2‐thienyl)naphthalene (**BTN**) (detailed procedures are summarized in the Supporting Information). After polymerization, the **PBTN** thin film was soaked in ethanol to remove residual iodine and monomers, resulting in a high‐purity and homogeneous **PBTN** thin film with a thickness of 8–30 nm (confirmed by energy‐dispersive X‐ray spectroscopy (**EDX**), X‐ray photoelectron spectroscopy (**XPS**), and scanning electron microscope (**SEM**), as shown in Figures S2–S4). The chemical structure of **PBTN** was confirmed by Raman spectroscopy (Figure S5 and Table S2). **PBTB** was also prepared by iodine‐vapor‐assisted polymerization, and its chemical structure was confirmed by **XPS** and Raman spectroscopy (Figures S3 and S5 and Table S3). The film thickness could be controlled by adjusting the concentration of the monomer solution and the spin‐coating speed.

The progress of polymerization was confirmed by matrix‐assisted laser desorption ionization‐time of flight mass spectroscopy (**MALDI**‐**TOF MS**) and Fourier transform infrared (**FT**‐**IR**) spectroscopy (Figure 1c,d). As shown in Figure 1c, the **MALDI**‐**TOF MS** spectrum indicated the formation of polymers comprising ≥20 **BTN** units. As shown in Figure 1d, the **FT**‐**IR** measurements demonstrated differences between the spectra of **PBTN** and **BTN** in the 600–800 cm^−1^ region, which corresponded to the C–H out‐of‐plane bending vibration (Table S4). In **BTN**, a strong absorption peak appeared at 696 cm^−1^, corresponding to the 2‐monosubstituted thiophene ring [43]. In contrast, in **PBTN**, the absorbance at 696 cm^−1^ decreased, and a new peak appeared at 796 cm^−1^, corresponding to the 2,5‐disubstituted thiophene ring. These results indicated the formation of **PBTN**.

The ultraviolet–visible spectroscopy (**UV**‐**Vis**) spectra (Figure 1e) showed that the HOMO/LUMO energy gap (*E*_g_), which was determined from the onset of absorption, was 2.42 and 2.19 eV for **PBTN** and **PBTB**. In addition, while **PBTB** exhibited multiple absorption peaks at 450, 490, and 540 nm due to intermolecular π‐stacking, **PBTN** exhibited only a single peak at 407 nm. This result supports the conclusion that the twist angle increased when the phenylene unit was replaced with a naphthalene unit, leading to weaker intermolecular π‐stacking interactions in **PBTN** than those in **PBTB**. The **UV**‐**Vis** spectra (Figure 1e) and atmospheric photoelectron spectroscopy results (Figure S6) showed that *E*_HOMO_ and *E*_LUMO_ of each π‐conjugated polymer were −3.19 and −5.61 eV for **PBTN** and −3.23 and −5.42 eV for **PBTB**, respectively (Figure 1f). Therefore, by replacing the phenyl unit of **PBTB** with a naphthalene unit, we succeeded in selectively deepening *E*_HOMO_ by 0.19 eV (Figure 1f).

### 2.2 Photo and Electrochemical Properties of PBTN Thin Film

First, we investigated the electrocatalytic ability of the **PBTN** thin film under dark conditions. As shown in Figure S7, similar to the **PBTB** thin films which we reported previously [29], **PBTN** thin films exhibited high electrocatalytic ability for the O_2_ reduction/H_2_O_2_ (HO_2_^−^) production reaction (Equation (2)) in alkaline aqueous solution at pH 12. In addition, as shown in Figure S8, this current almost disappeared under Ar bubbling, indicating that the **PBTN** thin film was reacting selectively with O_2_.

(1) $$O_{\text{2}}+2H^{+}+2e^{-}⇄H_{2}O_{2}\;\;\text{E}{}^{\circ}=0.695\;\text{V vs}.\;\text{SHE}\;\;\text{pH}\leq 11.6$$

(2) $$\begin{array}{c} O_{2}+H_{2}O+2e^{-}⇄{\text{HO}}_{2^{-}}+{\text{OH}}^{-}\;\;\text{E}{}^{\circ}=0.358\;\text{V vs}.\;\text{SHE}\\ \text{ pH}\;>\;11.6\;({\text{E}}_{\text{eq}}=-0.190\;\text{V vs}.\;\text{Ag}/\text{AgCl}\\ \text{ at pH }12) \end{array}$$

**FIGURE 2:**
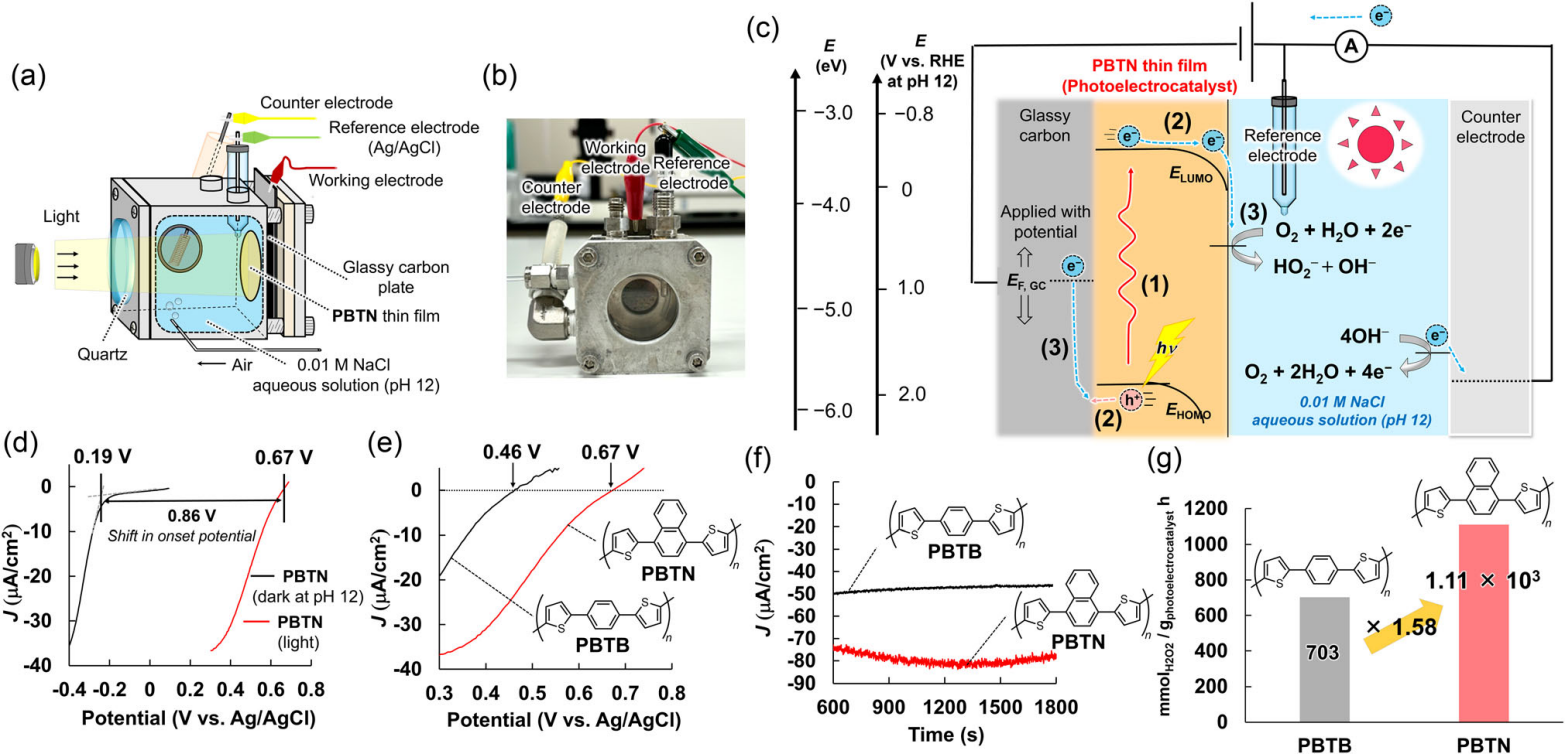
(a) Schematic of the photoelectrochemical cell setup. (b) Image of the photoelectrochemical cell setup. (c) Mechanism of photoelectrochemical H_2_O_2_ production by the PBTN thin film. (d) Linear sweep voltammograms (LSVs) recorded for the PBTN thin film (28 nm) as a cathode at 10 mV/s and pH 12 under air bubbling (4.0 mL/min). Black trace: under dark conditions; red trace: under visible‐light. The onset potential under visible‐light irradiation was defined as the point where the photocurrent curve intersects the *x*‐axis. Under dark conditions, the onset potential was defined as the falling edge of the reduction current in the LSV. (e) LSVs recorded for PBTN (28 nm, red trace) and PBTB (30 nm, black trace) thin films as cathodes under visible‐light at 10 mV/s and pH 12 under air bubbling (4.0 mL/min). (f) CA recorded for PBTN (28 nm, red trace) and PBTB (30 nm, black trace) thin films under 0 V vs. Ag/AgCl, air bubbling (200 mL/min) and visible‐light irradiation at pH 12. (g) Photoelectrocatalytic O_2_ reduction/H_2_O_2_ production rates of PBTN (8.0 nm) and PBTB (8.0 nm) thin films measured under 0 V vs. Ag/AgCl, air bubbling (200 mL/min) and visible‐light irradiation at pH 12.

Next, using the photoelectrochemical cell setup shown in Figure 2a,b, we demonstrated the photoelectrocatalytic ability of the **PBTN** thin films. The **PBTN** thin films are considered to produce H_2_O_2_ (HO_2_^−^ in the alkaline aqueous solution at pH 12) under visible‐light irradiation through the three steps as shown in Figure 2c. Specifically, (1) the **PBTN** thin films absorb visible‐light (Figure 1e) and generate a hole (h^+^)‐electron (e^−^) pair in the HOMO and LUMO. (2) The hole‐electron pair is separated; the electron diffuses to the surface of the **PBTN** thin film, while the hole diffuses to the glassy carbon (**GC**) substrate. (3) The electron is donated to O_2_, facilitating the reaction in Equation (2). The hole in the HOMO is neutralized by electron donation from the external circuit, and with an appropriate catalyst, the H_2_O oxidation/O_2_ production reaction in (Equation (3)) occurs at the counter electrode. For the external circuit to donate electrons to the HOMO, the potential applied to the **PBTN** thin films must be more negative than *E*_HOMO_. Therefore, *E*_HOMO_ is expected to significantly influence the onset potential.

(3) $$O_{\text{2}}+2H_{2}O+4e^{-}⇄4{\text{OH}}^{-}\;\;\text{E}{}^{\circ}=0.401\;\text{V vs}.\;\text{SHE}$$

As shown in Figure 2d,e, the onset potential for O_2_ reduction/H_2_O_2_ production of **PBTN** thin films under visible‐light irradiation was +0.67 V vs. Ag/AgCl (+1.56 V vs. RHE at pH 12), which was 0.21 V more positive than that of **PBTB** thin films (Table 1). Therefore, the difference in onset potential for light‐assisted O_2_ reduction/H_2_O_2_ production between **PBTN** and **PBTB** thin films (0.21 V, both approximately 30 nm thickness) closely matches the difference in their *E*_HOMO_ values (0.19 eV). This result indicates a strong correlation between *E*_HOMO_ of the π‐conjugated polymers and their onset potential for O_2_ reduction/H_2_O_2_ production under visible‐light irradiation. In addition, as shown in Figure 2f, chronoamperometry (**CA**) measurements were performed on **PBTN** and **PBTB** thin films with nearly identical thicknesses (30 nm) under visible‐light irradiation at 0 V vs. Ag/AgCl. The amount of H_2_O_2_ produced after the **CA** measurements was determined by spectrophotometric titration (Figure S9) [44]. The photoelectrocatalytic O_2_ reduction/H_2_O_2_ production rates for the **PBTN** and **PBTB** thin films were 468 ${\mathrm{mmol}}_{H_{2}O_{2}}$/g_photoelectrocatalyst_ h and 318 ${\mathrm{mmol}}_{H_{2}O_{2}}$/g_photoelectrocatalyst_ h, respectively (Table 1). Therefore, the photoelectrocatalytic O_2_ reduction/H_2_O_2_ production rate of the **PBTN** thin film was 1.47 times higher than that of the **PBTB** thin film. This is presumably because the more positive onset potential of the **PBTN** thin film compared with that of the **PBTB** thin film (Figure 2e) enables higher current density under the same potential of 0 V vs. Ag/AgCl. In addition, when the film thickness was adjusted to 8 nm, the **PBTN** thin films exhibited a high rate of O_2_ reduction/H_2_O_2_ production of 1.11 × 10^3^ ${\mathrm{mmol}}_{H_{2}O_{2}}$/g_photoelectrocatalyst_ h (37.7 $g_{H_{2}O_{2}}$/g_photoelectrocatalyst_ h) with a remarkably high Coulombic efficiency (99%) and selectivity (99%) under visible‐light irradiation, air bubbling, and 0 V vs. Ag/AgCl. This photoelectrocatalytic O_2_ reduction/H_2_O_2_ production rate was 1.58 times higher than that of the **PBTB** thin film (8 nm), which produced 703 ${\mathrm{mmol}}_{H_{2}O_{2}}$/g_photoelectrocatalyst_ h (23.9 $g_{H_{2}O_{2}}$/g_photoelectrocatalyst_ h), as shown in Figure 2g and Table 1. Furthermore, the photoelectrocatalytic O_2_ reduction/H_2_O_2_ production rate of **PBTN** thin film was higher than that reported in previous works [30, 45]. The higher photoelectrocatalytic performance observed in the thinner film is attributed to the homogeneity of the films prepared in this work, ensuring that the surface area did not decrease even when the film thickness was reduced. This was supported by electrochemical double‐layer capacitance measurements (Figure S10). In addition, **SEM** and Raman spectroscopy confirmed that the **PBTN** thin films did not degrade after the **CA** measurements (Figures S11 and S12). The energy levels and photoelectrocatalytic performances of **PBTN** and **PBTB** are summarized in Table 1.

**TABLE 1:** HOMO/LUMO energy levels and photoelectrocatalytic O_2_ reduction/H_2_O_2_ production rates of PBTN and PBTB.

**Polymer**	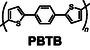	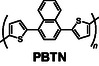
*E*_HOMO_ (eV)	−5.42	−5.61
*E*_LUMO_ (eV)	−3.23	−3.19
*E*_g_ (eV)	2.19	2.42
Onset potential (V vs. RHE at pH 12)	+1.35	+1.56
Photoelectrocatalytic O_2_ reduction/H_2_O_2_ production rate (30 nm) (${\mathrm{mmol}}_{H_{2}O_{2}}$/g_photoelectrocatalyst_ h)	318	468
Photoelectrocatalytic O_2_ reduction/H_2_O_2_ production rate (8 nm) (${\mathrm{mmol}}_{H_{2}O_{2}}$/g_photoelectrocatalyst_ h)	703	1.11 × 10^3^

### 2.3 Photocatalytic H_2_O_2_ Production by Combining PBTN Thin Film and Ni Foam

**FIGURE 3:**
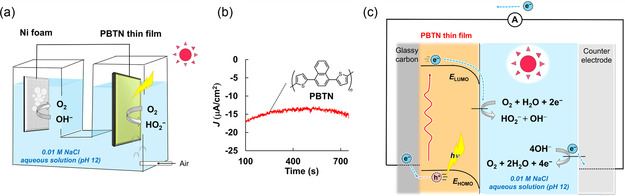
(a) Schematic of the full‐cell setup in the absence of a bias potential at pH 12 using a PBTN thin film (under visible‐light) and Ni foam as the cathode and anode. (b) CA recorded for the PBTN thin film (19 nm) in the absence of a bias potential under air bubbling (200 mL/min) and visible‐light at pH 12. (c) Mechanism of photocatalytic H_2_O_2_ production by PBTN.

Finally, as shown in Figure 3a, a full‐cell setup was fabricated using the **PBTN** thin film (19 nm) as the photocathode, a common H_2_O oxidation/O_2_ production electrocatalyst (Ni foam) as the anode [46], and 0.01 M NaCl aqueous solution (pH 12) as the electrolyte. Air bubbling and visible‐light irradiation on the photocathode enabled this setup to generate a reduction current (Figure 3b) and achieve a high photocatalytic O_2_ reduction/H_2_O_2_ production rate of 128 ${\mathrm{mmol}}_{H_{2}O_{2}}$/g_photocathode_ h (4.40 $g_{H_{2}O_{2}}$/g_photocathode_ h), which was 4.9 times higher than that obtained with **PBTB** thin film (22 nm). As shown in Figure 3c, the highly positive onset potential of the **PBTN** thin film for visible‐light‐assisted O_2_ reduction/H_2_O_2_ production (+1.56 V vs. RHE at pH 12) enabled efficient photocatalytic O_2_ reduction/H_2_O_2_ production without the application of a bias potential.

## 3 Conclusion

In this study, we found that replacing the phenyl unit of **PBTB**, which has previously been reported to exhibit high photoelectrocatalytic activity [29], with a naphthalene unit (a stronger electron‐withdrawing group) and increasing the twist angle of the polymer chain enabled *E*_HOMO_ to become selectively deeper than that of **PBTB**. **PBTN** thin films exhibited a more positive onset potential than that of **PBTB** thin film and achieved a high O_2_ reduction/H_2_O_2_ production rate of 1.11 × 10^3^ ${\mathrm{mmol}}_{H_{2}O_{2}}$/g_photoelectrocatalyst_ with a remarkably high Coulombic efficiency (99%) and selectivity (99%) under visible‐light irradiation and 0 V vs. Ag/AgCl. In addition, visible‐light irradiation enabled a full‐cell setup combining the **PBTN** thin film with a common H_2_O oxidation/O_2_ production electrocatalyst (Ni foam) to achieve a high photocatalytic O_2_ reduction/H_2_O_2_ production rate of 128 ${\mathrm{mmol}}_{H_{2}O_{2}}$/g_photocathode_ h. These results demonstrate the high tunability of the photoelectrocatalytic ability of π‐conjugated polymers through appropriate molecular design. We expect that π‐conjugated polymers exhibiting high H_2_O_2_ production rates, suitability for device fabrication, and high durability in previous reports [28, 29] will significantly advance the development of green and sustainable O_2_ reduction/H_2_O_2_ production technologies. In our ongoing work, we are planning to further enhance the photoelectrocatalytic activities of π‐conjugated polymers based on the design strategy presented in this study and to demonstrate continuous H_2_O_2_ production over extended periods, such as several months, using the device (or photocatalyst).

## Supporting Information

Additional supporting information can be found online in the Supporting Information section. The authors have cited additional references within the Supporting Information [31, 36, 43, 44, 47, 48]. **Supporting** **Scheme** **S1:** Synthesis of 1,4‐bis(2‐thienyl)naphthalene. **Supporting Fig. S1:** Most stable structures of BTN and BTB were calculated using DFT B3LYP/6‐31G. **Supporting Fig. S2**: **EDX** elemental analysis of **PBTN** thin film. **EDX** measurements gave only peaks assignable to C, S, and O with no peaks ascribable to residual oxidant (i.e., iodine) in the **PBTN** thin film (below the detection limit). **Supporting Fig. S3:** **XPS** measurements of (a) **PBTN** thin film and (b) **PBTB** thin film coated on glassy carbon. **XPS** measurements gave only peaks assignable to C, S, and O with no peaks ascribable to residual oxidant (i.e., iodine) in the **PBTN** and **PBTB** thin films (below the detection limit). **Supporting Fig. S4:** **SEM** images of **PBTN** film. **SEM** was taken on the **PBTN** film formed on the **GC** plate. The **PBTN** film exhibited a homogeneous surface structure on a 100 nm scale. **Supporting Fig. S5:** Raman spectra of (a) **PBTN** and (b) **PBTB** thin films. Raman laser wavelength is 785 nm. Detailed assignments are summarized in Table S2 and Table S3. **Supporting Fig. S6:** Photoelectron spectra of (a) **PBTN** and (b) **PBTB** measured by photoemission yield spectroscopy in air. The ionization potential was calculated by selecting a linearly arranged plot on the analysis software. In this work, the ionization potential was approximated as the HOMO energy level (*E*_HOMO_). **Supporting Fig. S7:** a, b) Linear sweep voltammograms (**LSVs**) recorded for **PBTN** as a cathode at 10 mV/s and different pHs. The electrocatalytic ability of **PBTN** was investigated under dark conditions and air bubbling. The **PBTN** thin film formed on **GC** plate was electrochemically tested at pH 2–12. The electrochemical response at pH 12 was clearly different from that at lower pH. **Supporting Fig. S8:** **LSV** recorded for **PBTN** as a cathode under dark conditions at 10 mV/s and pH 12. Dash trace: under Ar bubbling. Black trace: under air bubbling (4.0 mL/min). **Supporting Fig. S9:** Calibration plots for the determination of H_2_O_2_ concentration. (a) The calibration curve was created by plotting the absorption at 456 nm in the UV‐vis spectra. (b) UV‐vis spectra of solutions at different H_2_O_2_ concentrations. **Supporting Fig. S10:** Linear relationship between the scan rate and current density of **PBTN** thin film. Black: 8 nm, Gray: 30 nm. The capacitive currents were measured at 2.0 V vs. Ag/AgCl from cyclic voltammograms recorded in the non‐Faradaic potential range of 1.5 to 2.5 V vs. Ag/AgCl. The data points represent the average of the absolute values of anodic and cathodic current densities ((|*J*_a_| + |*J*_c_|)/2) at each scan rate. The electrochemical double‐layer capacitance (*C*_DL_) was determined from the slope of the linear fit. CDL is proportional to the electrochemically active surface area (**ECSA**) [4]. These results indicate that the **PBTN** thin film has a comparable or larger **ECSA** at 8 nm compared to that at 30 nm. This is presumably because thinner films are more susceptible to the minute irregularities of the substrate (glassy carbon) and this effect is thought to be the reason why the PBTN thin film (8 nm) has a larger **ECSA** than that of 30 nm. **Supporting Fig. S11:** **SEM** images of the **PBTN** thin film from the (a) the untested area and (b) area after **CA** measurement (under the same conditions as Figure 2f). The **SEM** images revealed no significant changes in the surface morphology of the **PBTN** thin film after the **CA** measurement. **Supporting Fig. S12:** Raman spectra of **PBTN** thin films before (black trace) and after (red trace) the **CA** measurement (under the same conditions as Figure 2f). The Raman spectra showed that the chemical structure of **PBTN** was maintained before and after the **CA** measurement. **Supporting Table S1:** HOMO/LUMO energy level calculated by DFT B3LYP/6‐31G. **Supporting Table S2:** Assignments of the Raman spectrum of **PBTN** [6, 7, 8, 9]. **Supporting Table** **S3** **:** Assignments of the Raman spectrum of **PBTB** [6, 9]. **Supporting Table S4:** Assignments of the **IR** spectra of **PBTN** and **BTN** [5, 6]. **Supporting Table S5:** Summary of solutions used for density tests. **Supporting Table S6:** Cartesian coordinates of the entry 1 monomer in Table S1. **Supporting Table S7:** Cartesian coordinates of the entry 2 monomer in Table S1. **Supporting Table S8:** Cartesian coordinates of the entry 3 monomer in Table S1. **Supporting Table S9:** Cartesian coordinates of the entry 4 monomer in Table S1. **Supporting Table S10:** Cartesian coordinates of the entry 5 monomer in Table S1. **Supporting Table S11:** Cartesian coordinates of the entry 6 monomer in Table S1. **Supporting Table S12:** Cartesian coordinates of the entry 7 monomer in Table S1. **Supporting Table S13:** Cartesian coordinates of the entry 8 monomer in Table S1. **Supporting Table S14:** Cartesian coordinates of the entry 9 monomer in Table S1. **Supporting Table S15:** Cartesian coordinates of the entry 10 monomer in Table S1.

## Funding

This study was supported by the Grants‐in‐Aid for Scientific Research (JP23K17945 (K. Oka), JP23H03827 (K. Oka), JP24K01552 (K. Oka), and JP25K21722 (K. Oka)) and Environment Research and Technology Development Fund (JPMEERF20241RA4, (K. Oka)).

## Conflicts of Interest

The authors declare no conflicts of interest.

## Supporting information

Supplementary Material

## Data Availability

The data that support the findings of this study are available from the corresponding author upon reasonable request.
