# Supplementary material for: Molecular Design Strategy of π‐Conjugated Polymers for Efficient Visible‐Light‐Driven Photoelectrocatalytic O2 Reduction to H2O2 Production
Source: ChemSusChem. 2026 Mar 1;19(5):e202502396. doi: 10.1002/cssc.202502396 (PMC12950356; doi:10.1002/cssc.202502396)
Supplement: Supplementary file 1 — Supplementary Material [file CSSC-19-e202502396-s001.pdf]

ChemSusChem

Supporting Information

Molecular Design Strategy of π-Conjugated Polymers for Efficient Visible-light-driven Photoelectrocatalytic O_2 Reduction to H_2O_2 Production

Riku Sawada,[a] Hitoshi Kasai,[a] Kouki Oka*[a, b, c]

[a] R. Sawada, Prof. Dr. H. Kasai, Prof. Dr. K. Oka
Institute of Multidisciplinary Research for Advanced Materials, Tohoku University
2-1-1 Katahira, Aoba-ku, Sendai, Miyagi 980-8577, Japan
E-mail: oka@tohoku.ac.jp (Kouki Oka)
[b] Prof. Dr. K. Oka
Carbon Recycling Energy Research Centre, Ibaraki University
4-12-1 Nakanarusawa, Hitachi, Ibaraki 316-8511, Japan
E-mail: oka@tohoku.ac.jp (Kouki Oka)
[c] Prof. Dr. K. Oka
Deuterium Science Research Unit, Centre for the Promotion of Interdisciplinary Education and Research, Kyoto University
Yoshida, Sakyo-ku, Kyoto 606-8501, Japan
E-mail: oka@tohoku.ac.jp (Kouki Oka)

Experimental section

Synthesis of 1,4-bis(2-thienyl)naphthalene

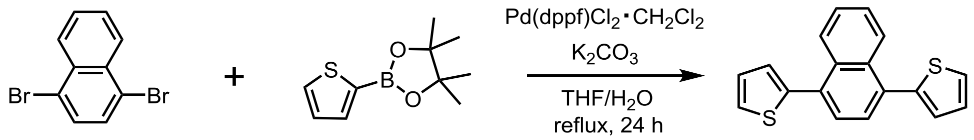

Scheme S1. Synthesis of 1,4-bis(2-thienyl)naphthalene.

1,4-Dibromonaphthalene (5.00 mmol, 1.00 eq), 2-(4,4,5,5-tetramethyl-1,3,2-diocaborolan-2- yl)thiophene (2.25 eq), and potassium carbonate (2.25 eq) were dissolved in a mixed solvent of THF and H_2O, and the mixture was then transferred to a dried two-necked recovery flask purged with pure nitrogen. [1,1'-Bis(diphenylphosphino)ferrocene]palladium(II) dichloride dichloromethane adduct (0.05 eq) was then added to the mixture under nitrogen. The resulting mixture was refluxed at 60°C for 24 h. The mixture was extracted with toluene and purified by silica gel column chromatography with toluene as the eluent and recrystallized to give a yellow powder. The powder was characterized as 1,4-bis(2-thienyl)naphthalene[1] (yield: 72%): ^{1}H NMR (400 MHz, chloroform-*d*, δ): 8.30–8.27 (dd, J = 9.00 Hz 2H), 7.59 (s, 2H), 7.54–7.51 (m, J = 9.86 Hz 2H), 7.47–7.45 (dd, J = 6.32 Hz 2H), 7.29–7.27 (dd, J = 4.68 Hz 2H), 7.22–7.20 (t, J = 8.60 Hz 2H).

Iodine Vapor-Assisted Polymerization to form PBTN Thin Film

A chlorobenzene solution of **BTN** was spin-coated onto a glassy carbon (**GC**), which was then placed in a pre-heated chamber with iodine at 90 °C for 1 h. The sample was soaked in ethanol repeatedly and dried in a vacuum chamber at 90 °C for 3 h.

Iodine Vapor-Assisted Polymerization to form PBTB Thin Film

1,4-Bis(2-thienyl)benzene (**BTB**) was prepared via the Suzuki–Miyaura coupling of 1,4-dibromobenzene and 2-(4,4,5,5-tetramethyl-1,3,2-diocaborolan-2-yl)thiophene, as described in the previous paper.[2] The **PBTB** thin film was synthesized in the same way as the **PBTN** thin film.

Photo-Electrochemical Testing

Illumination was provided by an Asahi Spectra MAX-303 300 W Xe lamp with an equivalent power of 1.0 SUN at the distance from the polymer plate (5 cm). The weight of the polymer layer specimen was determined by the layer area (2.5 cm^2), average thickness, and density of its fragments. (Details regarding the density test are provided in Table S5.)

In the full-cell setup, Ni foam containing no precious metals was used as the counter electrode. This measurement was performed for 15 minutes by setting the potential

between the Ni foam and the polymer plate to 0 V using the potentiostat (HZ-7000, HOKUTO DENKO).

H_2O_2 Quantification

The produced H_2O_2 by polymers was determined with spectrophotometric titration.[3] The H_2O_2 aqueous solutions of different concentrations used for the calibration curves were prepared by diluting a standard H_2O_2 aqueous solution (3 wt% without any additives, Millipore), and also adjusted to 0.01 M aqueous NaCl and pH 9 with NaCl, HCl, and NaOH. The calibration curve (Figure S9 (a)) was prepared based on the absorption at 456 nm of ultraviolet-visible spectroscopy (**UV-Vis**) spectra (Figure S9 (b)) of H_2O_2 aqueous solutions at different concentrations.

Supplementary methods

Substrate

GC substrates were cleaned by **UV** light for 30 min with ASM401oz (ASUMI GIKEN) prior to polymer formation. Glass plates were sonicated in chlorobenzene for 10 min prior to polymer formation. **GC** substrates, glass plates, and indium tin oxide (**ITO**) glass substrates were purchased from ALLIANCE Biosystems (Production code: 9999902-30304-22), AS ONE (Production code: 1-9645-11), and Shimadzu (Production code: 224-37183-58).

Electrochemical Testing

All measurements were performed using a photoelectrochemical cell that limited the contact area between the electrolyte and the polymer film, and the bubbling strength was adjusted using the flow meter (Azbil, F4Q9200B6TN100000). Ag/AgCl and Ti mesh were used as a reference and counter electrode, respectively. Measurements at different pHs were performed using the same sample. Aqueous solutions with different pH levels were prepared by adding aqueous NaOH or HCl to 0.01 M aqueous NaCl to maintain a moderate ionic strength for electrolysis. The pH of each electrolyte was tested with a pH meter (LAQUA, HORIBA). Air (a mixture of nitrogen and oxygen in atmospheric proportions) was bubbled through the cell for 30 min prior to testing.

Material Characterization

Matrix-assisted laser desorption ionization-time of flight mass spectroscopy (**MALDI-TOF MS**) (MALDI-8030, Shimadzu) was performed on the **PBTN** thin film formed on an **ITO** substrate. Scanning electron microscopy (**SEM**) images (accelerating voltage = 1.0 kV) and Energy dispersive X-ray spectrometry (**EDX**) (accelerating voltage = 15 kV) were obtained using JEOL Ltd. JSM-7800F. X-ray photoelectron spectroscopy (**XPS**) were obtained using a ULVAC-PHI PHI5000 VersaProbe II. Raman spectrum was obtained using a Jasco NRS-5500 with a 785 nm excitation laser. The **UV-Vis** absorption spectra of the polymer layers formed on grass plates were obtained using a Shimadzu UV-1900i. **UV-vis** absorption spectrum of the **PBTN** film measured in transmission mode. Fourier transform infrared (**FT-IR**) spectra of polymer fragments were obtained using Shimadzu **IR** spirit. The polymer layer thickness was measured using a KLA P-7.

Materials

1,4-Dibromonaphthalene (purity: >98%), 2-(4,4,5,5-tetramethyl-1,3,2-diocaborolan-2-yl)thiophene (purity: >98%), chlorobenzene (purity: >98%), and iodine (purity: >99%) were purchased from Tokyo Chemical Industry. Sodium hydroxide (NaOH) (purity: >97%), hydrochloric acid (HCl), and ethanol (purity: >99%) were purchased from FUJIFILM Wako Pure Chemical Corporation. Standard H_2O_2 solutions were purchased from Millipore and Sigma-Aldrich. Ni foam (Production code: 5203-ADE12) was purchased from Full Cell store.

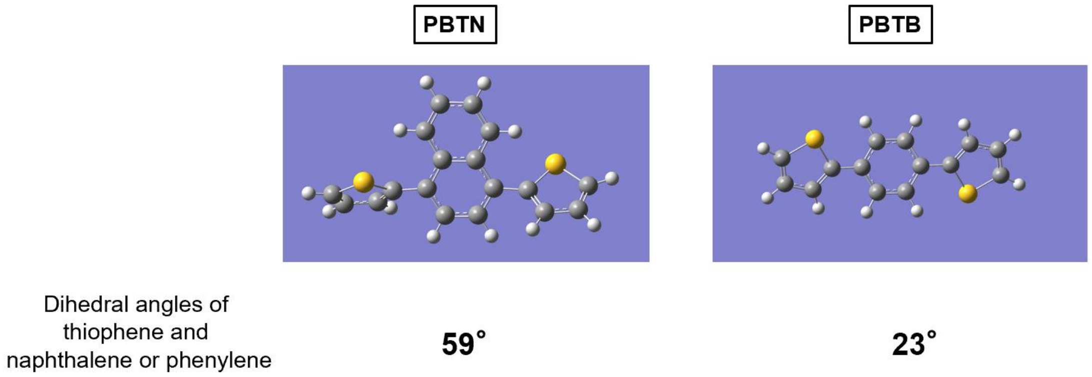

Figure S1. Most stable structures of BTN and BTB were calculated using DFT B3LYP/6-31G.

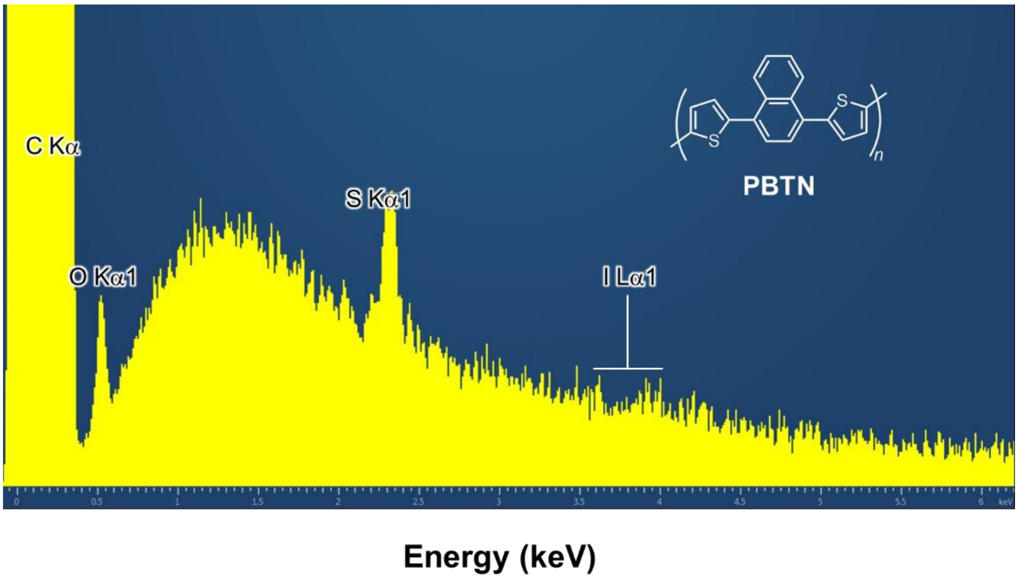

Figure S2. **EDX** elemental analysis of **PBTN** thin film. **EDX** measurements gave only peaks assignable to C, S, and O with no peaks ascribable to residual oxidant (i.e., iodine) in the **PBTN** thin film (below the detection limit).

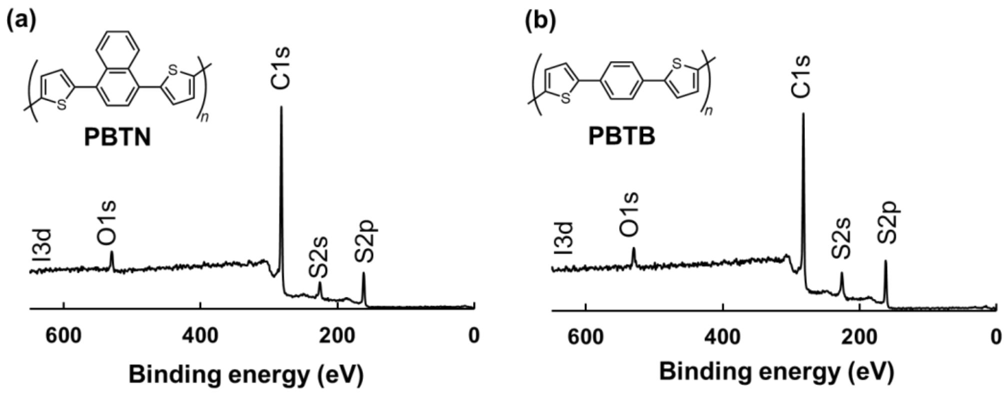

Figure S3. **XPS** measurements of (a) **PBTN** thin film and (b) **PBTB** thin film coated on glassy carbon. **XPS** measurements gave only peaks assignable to C, S, and O with no peaks ascribable to residual oxidant (i.e., iodine) in the **PBTN** and **PBTB** thin films (below the detection limit).

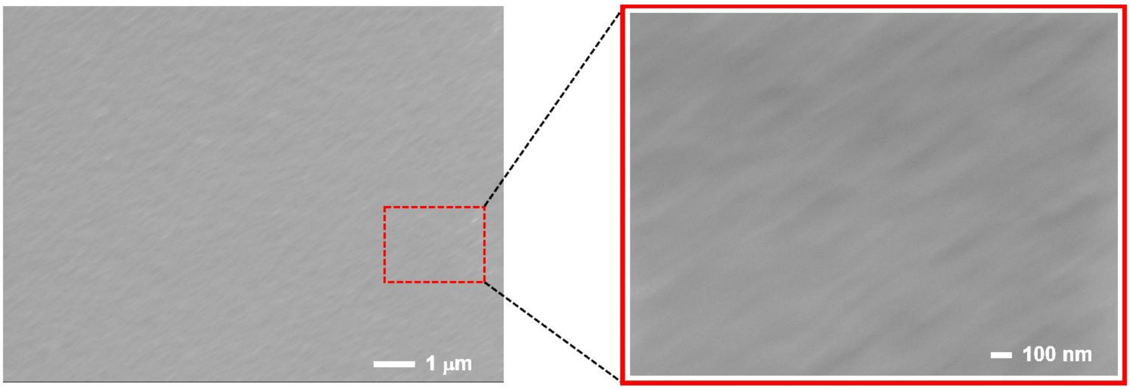

Figure S4. **SEM** images of **PBTN** film. **SEM** was taken on the **PBTN** film formed on the **GC** plate. The **PBTN** film exhibited a homogeneous surface structure on a 100 nm scale.

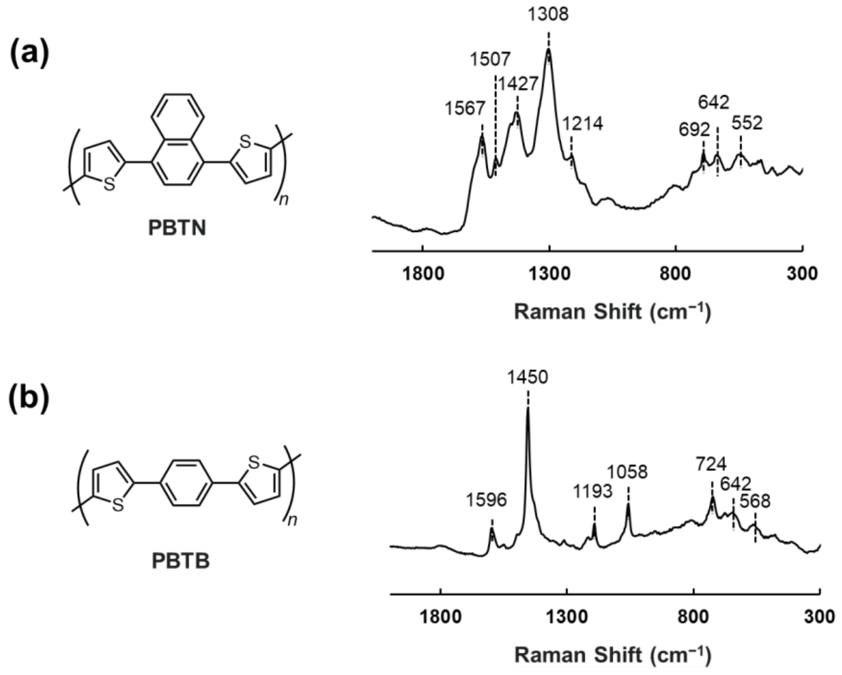

Figure S5. Raman spectra of (a) **PBTN** and (b) **PBTB** thin films. Raman laser wavelength is 785 nm. Detailed assignments are summarized in Table S2 and Table S3.

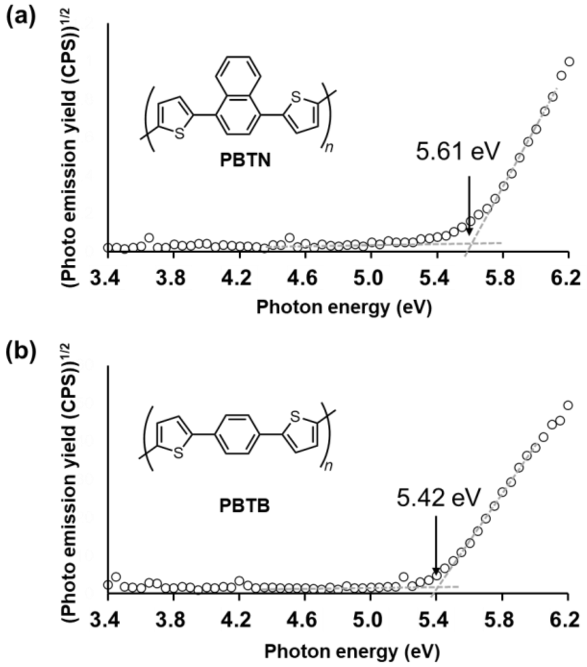

Figure S6. Photoelectron spectra of (a) **PBTN** and (b) **PBTB** measured by photoemission yield spectroscopy in air. The ionization potential was calculated by selecting a linearly arranged plot on the analysis software. In this work, the ionization potential was approximated as the HOMO energy level (E_{HOMO}).

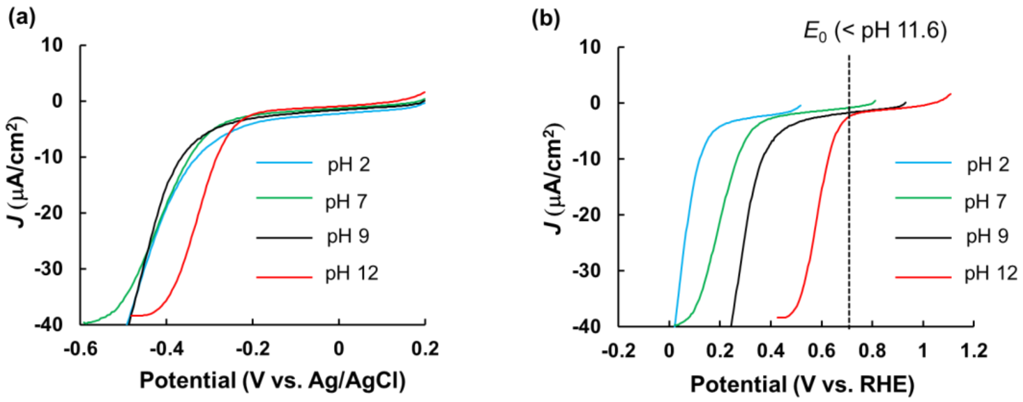

Figure S7. a, b) Linear sweep voltammograms (**LSVs**) recorded for **PBTN** as a cathode at 10 mV/s and different pHs. The electrocatalytic ability of **PBTN** was investigated under dark conditions and air bubbling. The **PBTN** thin film formed on **GC** plate was electrochemically tested at pH 2–12. The electrochemical response at pH 12 was clearly different from that at lower pH.

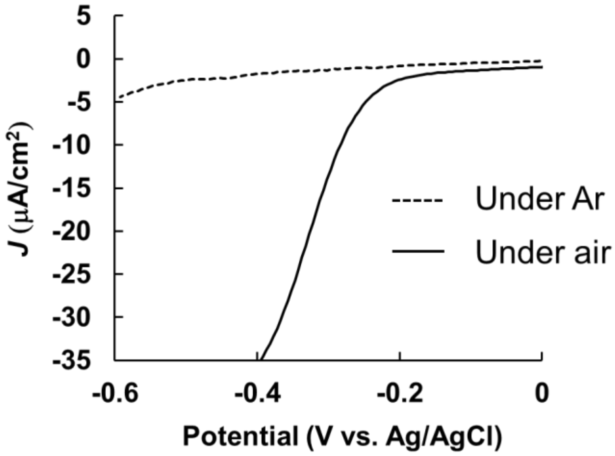

Figure S8. **LSV** recorded for **PBTN** as a cathode under dark conditions at 10 mV/s and pH 12. Dash trace: under Ar bubbling. Black trace: under air bubbling (4.0 mL/min).

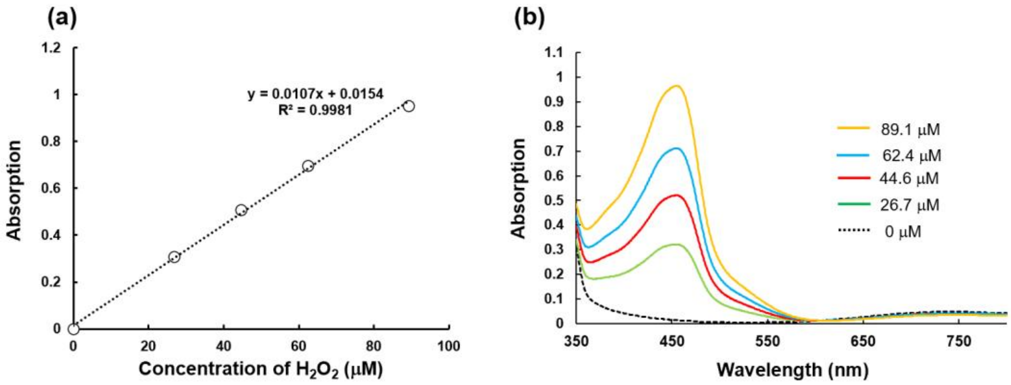

Figure S9. Calibration plots for the determination of H_2O_2 concentration. (a) The calibration curve was created by plotting the absorption at 456 nm in the UV-vis spectra. (b) UV-vis spectra of solutions at different H_2O_2 concentrations.

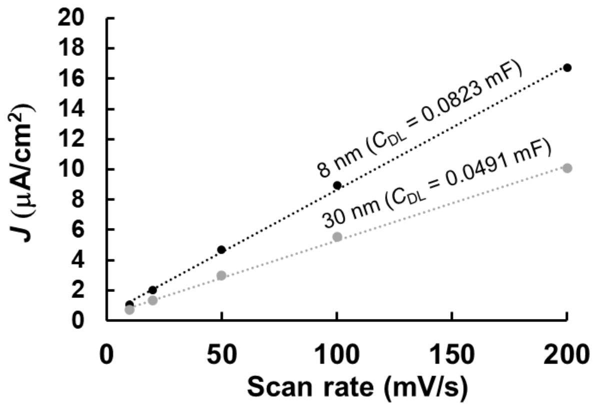

Figure S10. Linear relationship between the scan rate and current density of **PBTN** thin film. Black: 8 nm, Grey: 30 nm. The capacitive currents were measured at 2.0 V vs. Ag/AgCl from cyclic voltammograms recorded in the non-Faradaic potential range of 1.5 to 2.5 V vs. Ag/AgCl. The data points represent the average of the absolute values of anodic and cathodic current densities $((|J_a| + |J_c|)/2)$ at each scan rate. The electrochemical double-layer capacitance (C_{DL}) was determined from the slope of the linear fit. C_{DL} is proportional to the electrochemically active surface area (**ECSA**).[4] These results indicate that the **PBTN** thin film has a comparable or larger **ECSA** at 8 nm compared to that at 30 nm. This is presumably because thinner films are more susceptible to the minute irregularities of the substrate (glassy carbon) and this effect is thought to be the reason why the **PBTN** thin film (8 nm) has a larger **ECSA** than that of 30 nm.

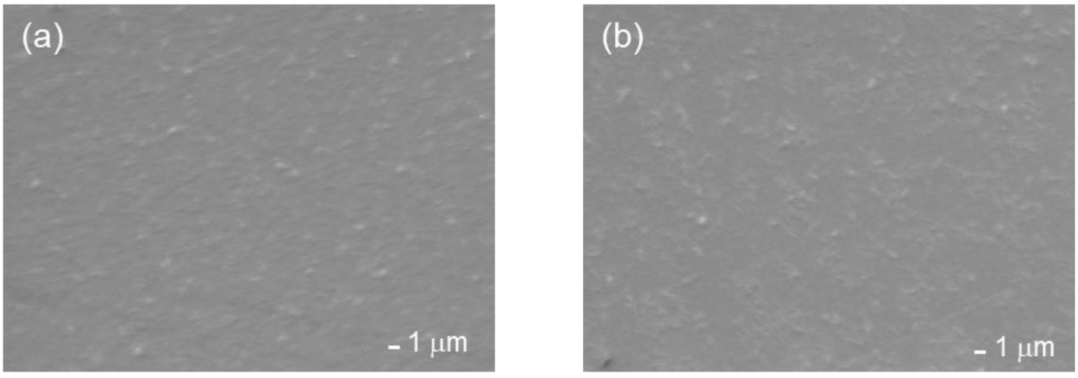

Figure S11. **SEM** images of the **PBTN** thin film from the (a) the untested area and (b) area after **CA** measurement (under the same conditions as Figure 2f). The **SEM** images revealed no significant changes in the surface morphology of the **PBTN** thin film after the **CA** measurement.

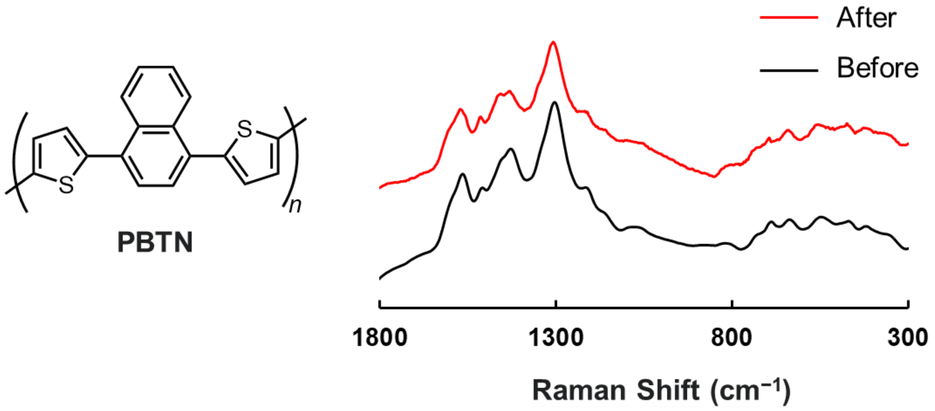

Figure S12. Raman spectra of **PBTN** thin films before (black trace) and after (red trace) the **CA** measurement (under the same conditions as Figure 2f). The Raman spectra showed that the chemical structure of **PBTN** was maintained before and after the **CA** measurement.

Table S1. HOMO/LUMO energy level calculated by DFT B3LYP/6-31G.

Entry	Monomer structure	E_{HOMO} (eV)	E_{LUMO} (eV)
1	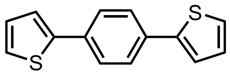	−5.51	−1.59
2	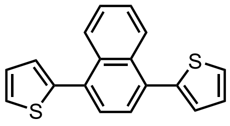	−5.59	−1.54
3	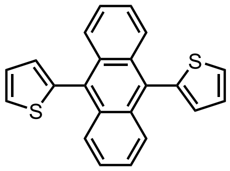	−5.34	−1.92
4	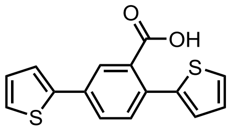	−5.87	−1.85
5	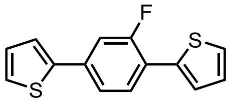	−5.63	−1.72
6	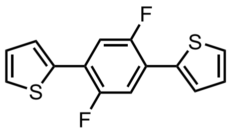	−5.74	−1.99
7	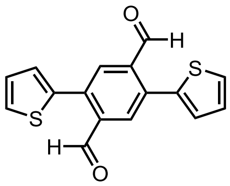	−6.22	−2.94
8	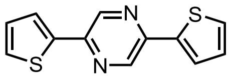	−5.99	−2.23
9	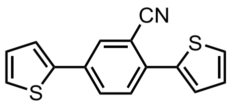	−5.96	−2.01
10	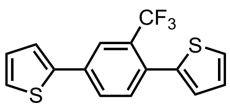	−6.00	−1.87

Table S2. Assignments of the Raman spectrum of **PBTN**. [6-9]

Assignment	**PBTN** (cm^{-1})
C– C– C ring breathing	552, 642
C_α–S–C_α stretching	692
C–H bending	1214
C–C stretching	1308
C_α=C_β symmetric vibrations (thiophene)	1427
C_α=C_β antisymmetric vibrations (thiophene)	1507
C=C Stretching (naphthalene)	1567

Table S3. Assignments of the Raman spectrum of **PBTB**. [6, 9]

Assignment	**PBTB** (cm^{-1})
C–C–C ring breathing	568, 642
C_α–S–C_α stretching	724
C–H bending (thiophene)	1058
C–H bending (phenylene)	1193
C_α=C_β symmetric vibrations (thiophene)	1450
C=C Stretching (phenylene)	1596

Table S4. Assignments of the **IR** spectra of **PBTN** and **BTN**.[5-6]

Assignment	**PBTN** (cm^{-1})	**BTN** (cm^{-1})
C–H out-of-plane bending vibration of 2,5-disubstituted thiophene ring	796	-
C– C– C ring breathing	764	765
C–H out-of-plane bending vibration of 2-monosubstituted thiophene ring	696	696

Table S5. Summary of solutions used for density tests.

Solution	Water : Ethanol	Density (g/cm^3)
A	9.5 : 0.5	0.989
B	9.4 : 0.6	0.986
C	9.3 : 0.7	0.984
D	9.2 : 0.8	0.980

Following previously reported methods,[16] we synthesized powders of **PBTN** and **PBTB**, and then performed density measurements using solutions A–D. Details of the four solutions prepared are shown in Table S5. As a result, **PBTN** powder diffused into solution C, and **PBTB** powder diffused into solution B. From these results, the density of **PBTN** was calculated to be 0.984 g/cm^3, and the density of **PBTB** was calculated to be 0.986 g/cm^3.

Cartesian coordinates of optimized geometries

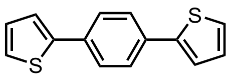

Table S6. Cartesian coordinates of the entry 1 monomer in Table S1.

Atom	X	Y	Z
C	0.60772	−1.21709	−0.29892
C	−0.77944	−1.11273	−0.30569
C	−1.4242	0.107465	−0.01125
C	−0.60772	1.217109	0.298962
C	0.779437	1.11274	0.305737
C	1.4242	−0.10745	0.011298
H	1.067969	−2.16289	−0.56308
H	−1.37285	−1.98161	−0.57065
H	−1.06797	2.162916	0.563077
H	1.372842	1.981621	0.570706
C	−2.87942	0.238272	−0.02869
C	−3.65045	1.368153	−0.16392
S	−3.95655	−1.22604	0.153696
C	−5.065	1.138431	−0.1321
H	−3.2207	2.351448	−0.31113
C	−5.40882	−0.17276	0.025699
H	−5.79643	1.931156	−0.23275
H	−6.38834	−0.62123	0.078857
C	2.879426	−0.23826	0.028736

C	3.650441	−1.36812	0.164184
S	3.956548	1.226006	−0.15394
C	5.065	−1.13842	0.132268
H	3.220705	−2.35138	0.311639
C	5.408832	0.172728	−0.02579
H	5.796414	−1.93114	0.23305
H	6.388344	0.62122	−0.07907

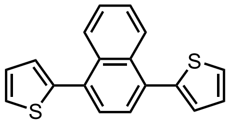

Table S7. Cartesian coordinates of the entry 2 monomer in Table S1.

Atom	X	Y	Z
C	0.700616	3.176668	0.098496
C	1.389237	1.982785	0.175144
C	0.718378	0.73048	0.067009
C	−0.7183	0.73062	−0.06692
C	−1.38892	1.983061	−0.17512
C	−0.70006	3.176798	−0.09865
H	1.235377	4.117022	0.185109
H	2.460964	1.990829	0.326286
C	1.429347	−0.52232	0.102527
C	−1.42943	−0.52209	−0.10244
H	−2.46067	1.991223	−0.32607
H	−1.23459	4.117269	−0.18543
C	−0.70311	−1.70553	−0.04179
C	0.702901	−1.70565	0.041887
H	−1.2346	−2.6508	−0.05165
H	1.234238	−2.65101	0.051746
C	2.894862	−0.61728	0.213962
C	3.621295	−1.34628	1.120399
S	4.022851	0.135013	−1.01773

C	5.041223	−1.33693	0.907301
H	3.148132	−1.87071	1.942116
C	5.430224	−0.6063	−0.17635
H	5.741033	−1.85649	1.550669
H	6.423392	−0.4261	−0.55739
C	−2.89492	−0.61715	−0.21392
C	−3.62126	−1.34637	−1.12025
S	−4.02312	0.135401	1.017562
C	−5.04121	−1.33714	−0.90719
H	−3.14794	−1.87088	−1.94183
C	−5.43032	−0.60635	0.176303
H	−5.74093	−1.85689	−1.55049
H	−6.42357	−0.42627	0.557189

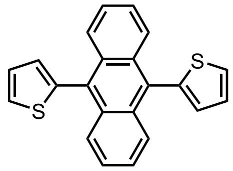

Table S8. Cartesian coordinates of the entry 3 monomer in Table S1.

Atom	X	Y	Z
C	0.710256	3.676602	−0.05644
C	1.400989	2.4909	−0.11072
C	0.721763	1.228868	−0.05689
C	−0.72172	1.228862	0.056593
C	−1.40098	2.490882	0.110331
C	−0.71027	3.676593	0.055891
C	1.426456	0.0003	−0.114
C	−1.42637	0.000284	0.113822
C	−0.72169	−1.22828	0.056764
C	0.721754	−1.22826	−0.05708
C	1.400951	−2.49027	−0.11163
H	2.480465	−2.4901	−0.2008
C	0.710259	−3.67599	−0.05703
C	−0.71018	−3.676	0.056453
C	−1.40088	−2.4903	0.111216
H	1.245078	4.620006	−0.09884
H	2.480571	2.490728	−0.19906
H	−2.48056	2.490689	0.198705
H	−1.24511	4.619993	0.098179

H	1.245048	−4.61938	−0.10002
H	−1.24496	−4.61941	0.099328
H	−2.48038	−2.49013	0.200431
C	2.901145	0.000348	−0.23181
C	3.683584	0.003215	−1.35418
S	3.957578	−0.0034	1.263433
C	5.100132	0.002629	−1.10377
H	3.256954	0.005692	−2.35034
C	5.424776	−0.00075	0.22036
H	5.841588	0.004612	−1.8937
H	6.399555	−0.00194	0.683017
C	−2.90105	0.000325	0.232043
C	−3.6834	0.003187	1.354503
S	−3.9579	−0.00339	−1.26305
C	−5.09997	0.002567	1.104341
H	−3.25662	0.005655	2.350598
C	−5.42481	−0.00077	−0.21975
H	−5.84129	0.004522	1.894387
H	−6.39969	−0.00195	−0.6822

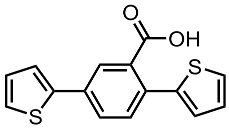

Table S9. Cartesian coordinates of the entry 4 monomer in Table S1.

Atom	X	Y	Z
C	−0.54676	0.824089	−0.19119
C	0.850163	0.922756	−0.11474
C	1.671891	−0.2107	0.023317
C	1.041719	−1.47001	0.067123
C	−0.34523	−1.57626	0.011959
C	−1.17786	−0.44628	−0.10599
H	1.296115	1.904101	−0.20185
H	1.638419	−2.36933	0.177568
H	−0.81207	−2.55133	0.092958
C	3.123991	−0.06261	0.113136
C	3.863272	1.039994	0.467224
S	4.235728	−1.45677	−0.27718
C	5.28272	0.841239	0.447531
H	3.407162	1.977052	0.762501
C	5.659248	−0.41788	0.079868
H	5.993313	1.616291	0.707431
H	6.649272	−0.83522	−0.0155
C	−2.63367	−0.66983	−0.10247
C	−3.36443	−1.46256	−0.94758

S	−3.701	−0.0046	1.216092
C	−4.75959	−1.55664	−0.62141
H	−2.92063	−1.95223	−1.80631
C	−5.11157	−0.85273	0.492447
H	−5.46852	−2.13037	−1.2059
H	−6.07925	−0.752	0.958631
C	−1.32086	2.057715	−0.46826
O	−2.46618	2.128965	−0.92613
O	−0.59824	3.211143	−0.1946
H	−1.14239	3.992265	−0.43201

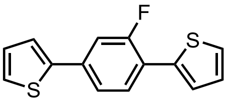

Table S10. Cartesian coordinates of the entry 5 monomer in Table S1.

Atom	X	Y	Z
C	0.490582	−1.37571	−0.26153
C	−0.89454	−1.24713	−0.26923
C	−1.51632	−0.0008	−0.04266
C	−0.67897	1.109976	0.200678
C	0.706149	0.981397	0.208378
C	1.327928	−0.26493	−0.01819
H	0.932855	−2.34311	−0.47288
H	−1.5044	−2.11897	−0.48196
H	−1.12124	2.077386	0.411982
C	−2.96907	0.154778	−0.06227
C	−3.72068	1.288694	−0.25905
S	−4.07099	−1.27726	0.207395
C	−5.1389	1.086472	−0.20903
H	−3.27435	2.25415	−0.46377
C	−5.50504	−0.20722	0.024684
H	−5.85669	1.885102	−0.35215
H	−6.49206	−0.63436	0.106933
C	2.780687	−0.42051	0.001419
C	3.532283	−1.55439	0.198413

S	3.882597	1.011466	−0.26853
C	4.950508	−1.3522	0.148297
H	3.085968	−2.5198	0.40337
C	5.316665	−0.05856	−0.08567
H	5.668293	−2.15082	0.291561
H	6.303674	0.368588	−0.16804
F	1.464932	2.066151	0.473062

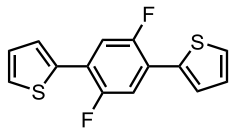

Table S11. Cartesian coordinates of the entry 6 monomer in Table S1.

Atom	X	Y	Z
C	0.585869	1.255586	0.072288
C	−0.78804	1.101858	0.074287
C	−1.44791	−0.13933	0.007507
C	−0.58587	−1.25558	−0.07228
C	0.788036	−1.10185	−0.07427
C	1.44791	0.139331	−0.00749
H	0.981484	2.259083	0.145791
H	−0.98149	−2.25908	−0.14579
C	−2.89534	−0.31401	0.025039
C	−3.59404	−1.49872	0.117241
S	−4.06764	1.080406	−0.10886
C	−5.01637	−1.3633	0.079447
H	−3.10903	−2.46119	0.221403
C	−5.44001	−0.07043	−0.04214
H	−5.69323	−2.20617	0.144792
H	−6.44749	0.312748	−0.08852
C	2.895341	0.314017	−0.02502
C	3.594046	1.498725	−0.11715
S	4.067641	−1.08041	0.108781

C	5.016376	1.363305	−0.07939
H	3.109033	2.461201	−0.22124
C	5.440013	0.070423	0.042101
H	5.693237	2.206174	−0.14469
H	6.447496	−0.31276	0.088428
F	1.546928	−2.27098	−0.15397
F	−1.54693	2.270985	0.153972

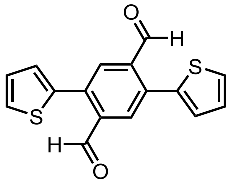

Table S12. Cartesian coordinates of the entry 7 monomer in Table S1.

Atom	X	Y	Z
C	0.613546	1.230988	0.030317
C	−0.7849	1.164395	0.14088
C	−1.43285	−0.09889	0.101047
C	−0.61353	−1.23084	−0.03048
C	0.784898	−1.16425	−0.14108
C	1.432871	0.099054	−0.10119
H	1.092653	2.203842	0.089365
H	−1.09266	−2.20368	−0.08951
C	−2.88982	−0.30896	0.174775
C	−3.57867	−1.05558	1.095213
S	−4.01613	0.293266	−1.12089
C	−4.98786	−1.15995	0.842399
H	−3.09438	−1.50504	1.954211
C	−5.38985	−0.51366	−0.28953
H	−5.66908	−1.6995	1.488746
H	−6.378	−0.43385	−0.71504
C	2.889841	0.309082	−0.17474
C	3.57889	1.055947	−1.09482
S	4.015954	−0.29372	1.120875

C	4.988071	1.160046	−0.84181
H	3.094781	1.505747	−1.95374
C	5.389851	0.513326	0.289936
H	5.669411	1.699736	−1.48791
H	6.377949	0.433267	0.715529
C	1.468062	−2.45524	−0.39429
O	2.660127	−2.61122	−0.68947
H	0.797513	−3.33247	−0.33124
C	−1.46808	2.455392	0.393949
O	−2.6602	2.611402	0.688889
H	−0.79751	3.332622	0.331019

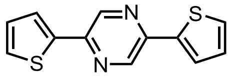

Table S13. Cartesian coordinates of the entry 8 monomer in Table S1.

Atom	X	Y	Z
C	−0.49408	−1.25284	−6.5E−05
C	−1.36503	−0.14236	−4.4E−05
C	0.494048	1.252759	−9.5E−05
C	1.365037	0.142234	−5.8E−05
H	−0.87139	−2.26905	−0.0001
H	0.871419	2.26894	−0.00014
C	−2.80798	−0.26688	0.000024
C	−3.60149	−1.39157	0.00021
S	−3.8255	1.238558	−0.00013
C	−5.00586	−1.11811	0.000203
H	−3.20123	−2.39819	0.000363
C	−5.30186	0.217057	0.00002
H	−5.76187	−1.89364	0.000326
H	−6.26994	0.693552	−3.6E−05
C	2.807962	0.266851	0.000014
C	3.601414	1.391581	0.000207
S	3.825567	−1.23852	−0.00014
C	5.005804	1.1182	0.00022
H	3.201126	2.398188	0.000337
C	5.301854	−0.21696	0.000037

H	5.761756	1.893786	0.000357
H	6.269959	−0.69341	0.000008
N	−0.83751	1.114525	−6.3E−05
N	0.837527	−1.11462	−6.1E−05

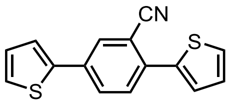

Table S14. Cartesian coordinates of the entry 9 monomer in Table S1.

Atom	X	Y	Z
C	0.430006	−1.41741	−0.18975
C	−0.95528	−1.30631	−0.21677
C	−1.5904	−0.05899	−0.04064
C	−0.76788	1.05902	0.180801
C	0.633512	0.954135	0.212781
C	1.270599	−0.30414	0.023305
H	0.888203	−2.3829	−0.36977
H	−1.55259	−2.1912	−0.40833
H	−1.21415	2.029405	0.36244
C	−3.04354	0.088699	−0.08761
C	−3.79502	1.212686	−0.33348
S	−4.13979	−1.33921	0.211321
C	−5.2125	1.004564	−0.29819
H	−3.35109	2.173769	−0.56299
C	−5.57485	−0.28308	−0.0274
H	−5.93206	1.793886	−0.47757
H	−6.56064	−0.71291	0.055381
C	2.71903	−0.51761	0.056276
C	3.380336	−1.60981	0.569677

S	3.913041	0.628603	−0.70655
C	4.800656	−1.58715	0.394021
H	2.862602	−2.40674	1.089982
C	5.252993	−0.48046	−0.266
H	5.457138	−2.36818	0.757221
H	6.263858	−0.20529	−0.52345
C	1.364551	2.153684	0.494398
N	1.899053	3.170425	0.738892

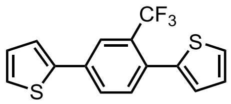

Table S15. Cartesian coordinates of the entry 10 monomer in Table S1.

Atom	X	Y	Z
C	0.189014	−1.70761	−0.11808
C	−1.19448	−1.56973	−0.15468
C	−1.7966	−0.30054	−0.06197
C	−0.94497	0.809716	0.091465
C	0.445013	0.674738	0.134529
C	1.054339	−0.60146	0.018057
H	0.630568	−2.69153	−0.22444
H	−1.81098	−2.45276	−0.28403
H	−1.37287	1.794961	0.216198
C	−3.24524	−0.117	−0.11849
C	−3.96668	1.017242	−0.40431
S	−4.37944	−1.50552	0.221989
C	−5.38911	0.847033	−0.36882
H	−3.49759	1.959283	−0.66082
C	−5.78564	−0.42121	−0.05743
H	−6.08728	1.648464	−0.57676
H	−6.78252	−0.8225	0.035285
C	2.498836	−0.88204	0.066597
C	3.109688	−1.84458	0.833053

S	3.721853	−0.09912	−1.03725
C	4.516986	−1.99091	0.60573
H	2.564516	−2.42514	1.567872
C	5.004296	−1.15258	−0.35455
H	5.136665	−2.69493	1.147351
H	6.014609	−1.04769	−0.71837
C	1.248179	1.92613	0.313686
F	1.878434	2.330403	−0.86978
F	0.459466	3.008023	0.707257
F	2.247046	1.804109	1.272875

【References】

[1] A. M. Fraind, J. D. Tovar, *The Journal of Physical Chemistry B* **2010**, *114*, 3104-3116.

[2] K. Oka, K. Noguchi, T. Suga, H. Nishide, B. Winther-Jensen, *Advanced Energy Materials* **2019**, *9*, 1803286.

[3] A. N. Baga, G. R. A. Johnson, N. B. Nazhat, R. A. Saadalla-Nazhat, *Analytica Chimica Acta* **1988**, *204*, 349-353.

[4] C. C. L. McCrory, S. Jung, I. M. Ferrer, S. M. Chatman, J. C. Peters, T. F. Jaramillo, *Journal of the American Chemical Society* **2015**, *137*, 4347-4357.

[5] S. Venkatachalam, D. R. Karunathan, V. Kannappan, *Journal of Chemistry* **2013**, *2013*.

[6] M. Akimoto, Y. Furukawa, H. Takeuchi, I. Harada, Y. Soma, M. Soma, *Synthetic Metals* **1986**, *15*, 353-360.

[7] J. P. Susairaj, S. Kaya, R. Ramamoorthy, E. Teju, B. Maria Susai, *Chemistry Africa* **2020**, *3*, 371-390.

[8] G. Louarn, M. Trznadel, J. P. Buisson, J. Laska, A. Pron, M. Lapkowski, S. Lefrant, *The Journal of Physical Chemistry* **1996**, *100*, 12532-12539.

[9] G. Louarn, M. Lapkowski, S. Quillard, A. Pron, J. P. Buisson, S. Lefrant, *The Journal of Physical Chemistry* **1996**, *100*, 6998-7006.

[10] D. Seo, V. Somjit, D. H. Wi, G. Galli, K.-S. Choi, *Journal of the American Chemical Society* **2025**, *147*, 3261-3273.

[11] D. Seo, A. Grieder, A. Radmilovic, S. F. Alamudun, X. Yuan, Y. Ping, K.-S. Choi, *Journal of Materials Chemistry A* **2024**, *12*, 20437-20448.

[12] S. Moon, Y. S. Park, H. Lee, W. Jeong, E. Kwon, J. Lee, J. Yun, S. Lee, J. H. Kim, S. Yu, J. Moon, *Energy & Environmental Science* **2024**, *17*, 5588-5600.

[13] K. Oka, K. Kamimori, B. Winther-Jensen, H. Nishide, *Advanced Energy and Sustainability Research* **2021**, *2*, 2100103.

[14] Z. Zhang, X. Chen, R. Hao, Q. Feng, E. Xie, *Advanced Functional Materials* **2023**, *33*, 2303391.

[15] M. Gryszel, A. Markov, M. Vagin, E. D. Głowacki, *Journal of Materials Chemistry A* **2018**, *6*, 24709-24716.

[16] K. Okubo, S. Kitajima, H. Kasai, K. Oka, *Small* **2025**, *21*, 2410794.
